# Immunoenhancing Effects of Jeju *Agastache rugosa* Extracts on NK Cell Activity and Lymphocyte Proliferation in Cyclophosphamide-Induced Immunosuppression

**DOI:** 10.3390/ph18081129

**Published:** 2025-07-28

**Authors:** Sung Jin Kim, Seong-Il Kang, Nari Lee, Jung Min Oh, Hiyoung Kim, Mi-Gi Lee, Ji Hoon Song, Myoung-Sook Shin

**Affiliations:** 1 College of Korean Medicine, Gachon University, Seongnam 13120, Republic of Korea; 2 Jeju Institute of Korean Medicine, Jeju-si 63309, Republic of Korea; 3 Department of Biomedical Science and Engineering, Konkuk University, Seoul 05029, Republic of Korea; 4 Bio-Center, Gyeonggi-do Business and Science Accelerator, Suwon 16229, Republic of Korea; 5 Bioindex, Seoul 02091, Republic of Korea

**Keywords:** *Agastache rugosa*, immunoenhancement, cyclophosphamide, NK cell activity, IFN-γ, splenic lymphocyte

## Abstract

**Background/objectives:** This study evaluated the immunoenhancing effects of *Agastache rugosa* extract in a cyclophosphamide-induced immunosuppressed mouse model. **Methods:** Jeju *A. rugosa* was processed via hot water extraction and 20% ethanol extraction. For immunosuppression induction, 7-week-old male BALB/c mice received intraperitoneal CPA injections (150 mg/kg, day −3; 110 mg/kg, day −1), followed by oral administration of hot water extract (ARE-W) and ethanol extract (ARE-E) at 100 and 300 mg/kg for 14 days. Oral administration of ARE-W and ARE-E was started on day 0, immediately following the final CPA injection on day −1. Immune function was assessed through body weight changes, spleen weight, NK cell activity, IFN-γ production, and splenic lymphocyte proliferation. **Results:** Results demonstrated that CPA treatment induced comprehensive immune dysfunction, while *A. rugosa* extracts significantly ameliorated these immunosuppressive conditions. Notably, ARE-W (300 mg/kg) significantly enhanced NK cell cytotoxicity against tumor cells and IFN-γ production compared to the CPA group, and effectively restored spleen weight and lymphocyte proliferation. ARE-E also exhibited dose-dependent immune function recovery; however, ARE-W showed superior efficacy across most immune parameters. **Conclusions:** These findings suggest that *A. rugosa* extract, particularly ARE-W, effectively restores immune function in immunosuppressed conditions, indicating potential application as a natural functional material for ameliorating immunosuppression caused by cancer treatment or immune diseases.

## 1. Introduction

The immune system is an essential mechanism that defends the body against various pathogenic microorganisms and harmful substances, composed of innate and adaptive immunity [1,2]. Normal immune function plays a critical role in the defense against infectious diseases as well as in maintaining homeostasis against cancer and autoimmune diseases [3,4]. However, immune function can be compromised by various factors, including disease, drug treatment, stress, and nutritional imbalance. Such immunosuppressive states can lead to increased susceptibility to infection, delayed disease recovery, and chronic inflammatory conditions, causing serious health problems [5]. In modern medicine, various synthetic immunomodulators are used to treat immunosuppressive states, but they often accompany serious side effects and present problems such as the development of resistance and increased risk of secondary infection with long-term use [6,7]. Due to these limitations, an interest in developing immunoenhancing materials from natural products has increased, and research on natural substances with effective immunomodulatory activity with fewer side effects is actively being conducted [8,9,10].

Cyclophosphamide (CPA) is widely used in clinical settings as an anticancer agent and immunosuppressant, with a mechanism for inhibiting cell proliferation through DNA alkylation [11]. CPA is an alkylating agent used for treating autoimmune diseases such as systemic lupus erythematosus, nephritis, multiple sclerosis, and rheumatoid arthritis [12]. CPA metabolites alkylate the DNA of splenic cells, suppressing immune responses by T and B lymphocytes [13]. Due to these characteristics, CPA is widely used in establishing immunosuppressed animal models and is recognized as a useful experimental model for evaluating the efficacy of immunoenhancing substances [14].

*Agastache rugosa* is a perennial herbaceous plant belonging to the Lamiaceae family, native to East Asian regions, including Korea, China, and Japan, and has been widely used in traditional medicine for aromatic, stomachic, antipyretic, and analgesic applications, among others [15]. *A. rugosa* has a distinctive aroma, and its main bioactive components include essential oils, flavonoids, and phenolic compounds [16,17,18]. These components have been reported to exhibit various pharmacological activities, including antioxidant, anti-inflammatory, antimicrobial, and antiviral effects [19,20,21]. Recent studies have shown that *A. rugosa* extract is also effective in improving cognitive function and gastric inflammation [22,23].

Natural killer (NK) cells play an important role in the innate immune system, with functions to directly recognize and eliminate virus-infected and tumor cells, and regulate interconnected immune responses through the secretion of cytokines such as interferon-gamma (IFN-γ) [24]. The impairment of NK cell activity leads to weakened host defense against infection and tumors, and recovering this function in immunosuppressed states is an important strategy for enhancing the immune system [25].

The bioactivity of plant extracts can vary significantly depending on the extraction solvent and method. Generally, hot water extraction tends to extract polar compounds, while organic solvent extraction selectively extracts non-polar or medium-polar compounds [26,27,28]. Therefore, even with the same plant material, the composition and content of bioactive substances can differ according to the extraction method, resulting in differences in biological efficacy. Particularly, the development of natural materials that are effective in restoring the immune function in immunosuppressed states has significant clinical implications for managing immunosuppressive conditions arising from cancer treatment, organ transplantation, and autoimmune disease therapy [29,30,31,32,33]. In this study, we aimed to compare the immunoenhancing effects of hot water extract (ARE-W) and 20% ethanol extract (ARE-E) of *A. rugosa* in a CPA-induced immunosuppressed mouse model. Specifically, we comprehensively analyzed various immunological parameters, including body weight changes, spleen weight, NK cell activity, IFN-γ production, and splenic lymphocyte proliferation, to elucidate the immunomodulatory efficacy and action characteristics of *A. rugosa* extracts. The results of this study are expected to provide scientific evidence for the potential application of *A. rugosa* as a natural functional material for enhancing immune function in immunosuppressed states.

## 2. Results

### 2.1. Effect of ARE-W and ARE-E on Body Weight Changes in Mice

**Figure 1:**
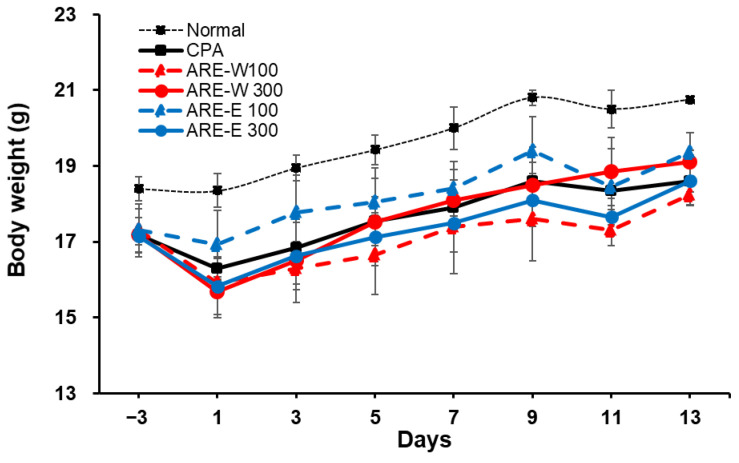
Effect of ARE-W and ARE-E on body weight in cyclophosphamide-induced immunosuppressed mice. BALB/c mice received CPA injections (150 mg/kg, day −3; 110 mg/kg, day −1) or PBS (normal). Mice were then orally administered with ARE-W or ARE-E (100 or 300 mg/kg) or vehicle daily for 14 days. Body weights were measured every 2 days.

Body weight recovery can be interpreted as a result of the combined effects of immune function improvement and metabolic activity promotion by *A. rugosa* extract, representing an important indicator of overall health status improvement in immunosuppressed states. Body weight change is an important indicator reflecting immune function and overall health status. In this study, we evaluated the effect of *A. rugosa* extract administration on body weight recovery in CPA-induced immunosuppressed states. The results in Figure 1 showed that the CPA group exhibited marked body weight reduction starting from day 1 of administration, which is considered to be due to CPA’s immunotoxicity and metabolic dysfunction. The normal group showed a steady weight gain trend throughout the experimental period, while the CPA group maintained significantly lower body weight compared to the normal group throughout the 14-days experimental period.

In the extract-administered groups, both ARE-W and ARE-E showed dose-dependent body weight recovery effects. Particularly, the ARE-W 300 mg/kg group showed significant body weight gain compared to the CPA group from day 5 of administration, and by the end of the experiment, the body weight recovered to approximately 92% of the normal group. The ARE-E 300 mg/kg group also showed a similar weight recovery pattern and ultimately achieved comparable levels of body weight recovery as the ARE-W 300 mg/kg group. The low-dose groups (ARE-W 100 mg/kg, ARE-E 100 mg/kg) showed relatively modest body weight recovery effects compared to the high-dose groups; however, gradual improvement was observed throughout the latter part of the experiment (Figure 1).

These results suggest that *A. rugosa* extract, particularly ARE-W, can effectively improve body weight reduction in CPA-induced immunosuppressed states. Body weight recovery can be interpreted as a result of the combined effects of immune function improvement and metabolic activity promotion by *A. rugosa* extract, representing an important indicator of overall health status improvement in immunosuppressed states.

### 2.2. Effect of ARE-W and ARE-E on Spleen Weight Changes in Mice

The spleen is a major immune organ that plays a critical role in lymphocyte maturation, differentiation, and immune response regulation. In this study, we analyzed the effect of *A. rugosa* extract on spleen weight in CPA-induced immunosuppressed states.

**Figure 2:**
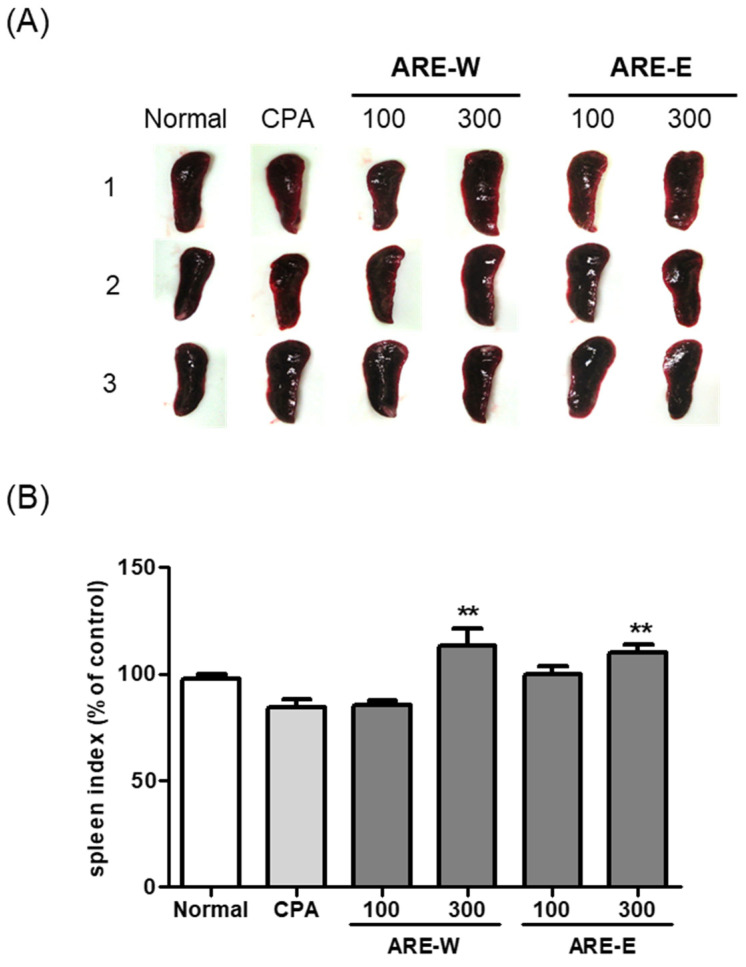
Effect of ARE-W and ARE-E on the spleen index in BALB/c mice: (**A**) representative images of spleens from each experimental group, with three animals per group; (**B**) quantification of spleen weight expressed as spleen index (mg spleen weight/g body weight) and presented as percentage of control (normal group). Data are presented as mean ± SD (*n* = 3). Statistical significance was determined by one-way ANOVA followed by Tukey’s post-hoc test. ** *p* < 0.01 versus the CPA group.

The results in Figure 2 showed that the CPA-treated group had a significant 19% decrease in spleen weight compared to the normal group. This reduction in spleen weight reflects lymphocyte cell death and proliferation inhibition due to the cytotoxic effect of CPA, which can be interpreted as a clear indicator of immune function impairment. Administration of *A. rugosa* extract showed significant recovery effects on spleen weight. Particularly, the ARE-W (300 mg/kg) group exhibited a remarkable 40% increase in spleen weight compared to the CPA group (*p* < 0.001), showing recovery close to the normal group level. The ARE-E (300 mg/kg) group also showed a significant 22% increase in spleen weight compared to the CPA group (*p* < 0.001). The low-dose groups (ARE-W 100 mg/kg, ARE-E 100 mg/kg) also showed trends of spleen weight increase, but the effects were relatively weaker compared to the high-dose groups and did not reach statistical significance compared to the CPA group.

When comparing effects according to the extraction method, ARE-W was more effective than ARE-E in restoring spleen weight at the same concentration (300 mg/kg). This suggests that bioactive substances with immunoenhancing activity might be more efficiently extracted during the hot water extraction process.

### 2.3. Effect of Oral Administration of ARE-W and ARE-E on NK Cell Activity in Mice

Natural killer (NK) cells are essential components of the innate immune system that play a crucial role in directly recognizing and eliminating virus-infected and tumor cells. In this study, we evaluated the effect of *A. rugosa* extract on NK cell cytotoxic activity in a CPA-induced immunosuppressed mouse model.

**Figure 3:**
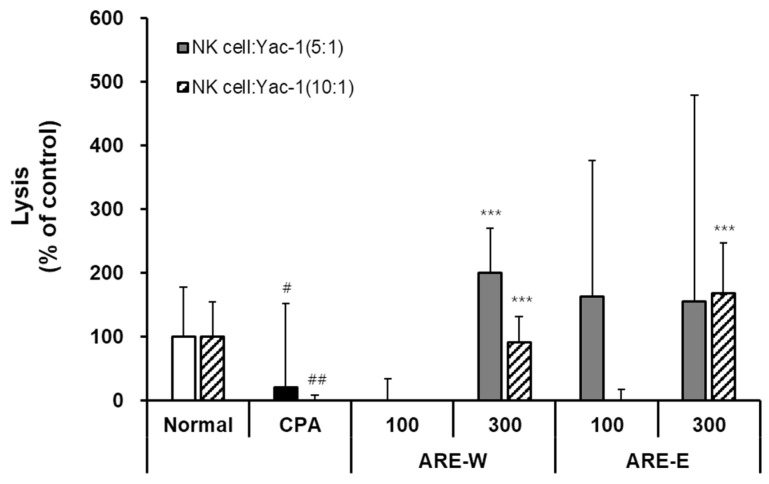
Effect of ARE-W and ARE-E on NK cell activity in CPA-treated immunosuppressed mice. The CPA and extract administration groups received intraperitoneal injections of cyclophosphamide (150 mg/kg, day −3; 110 mg/kg, day −1), followed by daily oral administration of ARE-W or ARE-E (100 or 300 mg/kg) for 14 days. The group treated with CPA alone served as the control group. NK cells isolated from spleens of different experimental groups were incubated with YAC-1 target cells at effector:target ratios of 5:1 (solid bars) and 10:1 (hatched bars) for 6 h at 37 °C, 5% CO_2_. Cytotoxic activity was determined by measuring LDH release in culture supernatants and calculated as the percentage of specific lysis. Data are presented as mean ± SD (*n* = 3). Statistical significance was determined by one-way ANOVA followed by Tukey’s post-hoc test. # *p* < 0.05, ## *p* < 0.01 versus the normal and *** *p* < 0.001 versus the CPA group.

The results in Figure 3 showed that CPA administration significantly reduced NK cell cytotoxicity against tumor cells (YAC-1) compared to the normal group. NK cells from the normal group exhibited stable cytotoxic activity at both 5:1 and 10:1 effector:target (E:T) ratios, whereas the CPA group showed markedly reduced activity to about 20% of normal group levels at both ratios. This clearly demonstrates the functional impairment of NK cells due to CPA’s immunosuppressive effect. Administration of *A. rugosa* extract significantly restored CPA-inhibited NK cell activity. Notably, the ARE-W (300 mg/kg) group showed an approximately 200% increase in NK cell activity compared to the CPA group at an E:T ratio of 5:1, exceeding the activity level of the normal group. A similar pattern of activity increase was observed at an E:T ratio of 10:1. The ARE-E groups also showed significant increases in NK cell activity. Both ARE-E 100 mg/kg and 300 mg/kg groups exhibited significantly higher NK cell activity compared to the CPA group, and particularly at an E:T ratio of 10:1, the 300 mg/kg group showed activity recovery similar to the normal group level. Analyzing dose dependence, both ARE-W and ARE-E showed higher NK cell activity at 300 mg/kg than at 100 mg/kg, suggesting that the NK cell activating effect of *A. rugosa* extract is dose-dependent. When comparing effects according to the extraction method, ARE-W was more effective than ARE-E in enhancing NK cell activity at the same concentration (300 mg/kg). This difference suggests that specific bioactive substances involved in NK cell activation might be more efficiently extracted during the hot water extraction process. These results demonstrate that *A. rugosa* extract, particularly ARE-W, can effectively restore NK cell cytotoxic function in CPA-induced immunosuppressed states. Since NK cells play a crucial role in virus infection and tumor surveillance, this NK cell activity-enhancing effect suggests the potential clinical application of *A. rugosa* extract in cancer therapy or immunodeficiency diseases.

### 2.4. Effect of Oral Administration of ARE-W and ARE-E on IFN-γ Production in Mice

**Figure 4:**
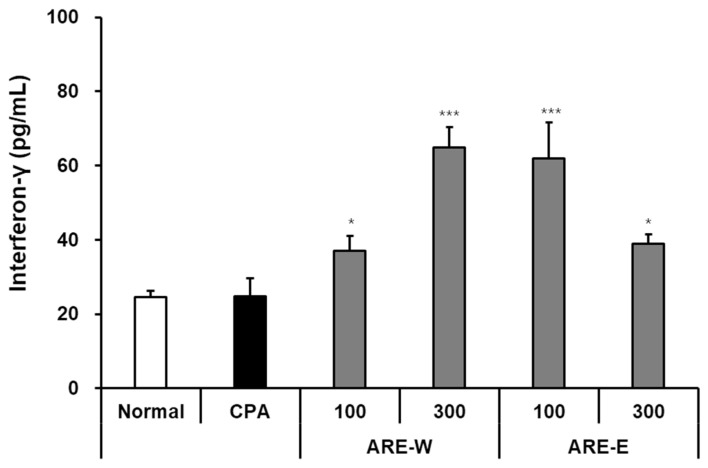
Effect of IFN-γ production by NK cells from mice administered different types of ARE-W and ARE-E. The CPA and extract administration groups received intraperitoneal injections of cyclophosphamide (150 mg/kg, day −3; 110 mg/kg, day −1), followed by daily oral administration of ARE-W or ARE-E (100 or 300 mg/kg) for 14 days. The group treated with CPA alone served as the control group. NK cells isolated from spleens of different experimental groups were co-incubated with YAC-1 cells for 20 h. The concentration of IFN-γ in culture supernatants was measured using an ELISA kit and expressed as pg/mL. Data are presented as mean ± SD (n = 3). Statistical significance was determined by one-way ANOVA followed by Tukey’s post-hoc test. * *p* < 0.05 and *** *p* < 0.001 versus the CPA group.

Interferon-gamma (IFN-γ) is a key cytokine secreted by NK cells and T cells, serving as an important mediator connecting innate and adaptive immunity. In this study, we quantitatively evaluated the effect of *A. rugosa* extract on IFN-γ production by NK cells in a CPA-induced immunosuppressed mouse model using the ELISA method. The results in Figure 4 showed that NK cells from the normal group secreted 24.5 ± 2.1 pg/mL of IFN-γ after co-culture with YAC-1 cells, while the CPA group secreted 23.2 ± 3.4 pg/mL, not showing a significant difference. However, considering that the tumor cell killing ability of NK cells was markedly decreased in immunosuppressed states, it can be concluded that NK cells in the CPA group had impaired tumor cell recognition and killing ability, but the function of IFN-γ secretion itself after activation was relatively preserved. Administration of *A. rugosa* extract significantly increased IFN-γ production by NK cells. Particularly, the ARE-W (300 mg/kg) group showed 65.2 ± 5.8 pg/mL of IFN-γ secretion, an approximately 2.8-fold increase compared to the CPA group, which was statistically highly significant. The ARE-W 100 mg/kg group also showed a significant increase to 37.6 ± 3.2 pg/mL, confirming a dose-dependent effect. The ARE-E groups also showed increased IFN-γ production. The ARE-E 100 mg/kg group showed 61.5 ± 8.4 pg/mL, an approximately 2.7-fold increase compared to the CPA group, and the 300 mg/kg group showed a significant increase to 39.2 ± 2.6 pg/mL. Notably, the ARE-E group showed a higher IFN-γ production-promoting effect at a low dose (100 mg/kg) than at a high dose (300 mg/kg). The increase in IFN-γ production by NK cells has important immunological implications. IFN-γ strengthens antiviral and antitumor immunity through various mechanisms, including macrophage activation, increased MHC molecule expression, and promotion of Th1-type immune responses. Therefore, the increased IFN-γ production by *A. rugosa* extract suggests its potential to induce broad activation across the immune system, in addition to enhancing the direct cytotoxicity of NK cells.

### 2.5. Effect of Oral Administration of ARE-W and ARE-E on Splenic Lymphocyte Proliferation in Mice

**Figure 5:**
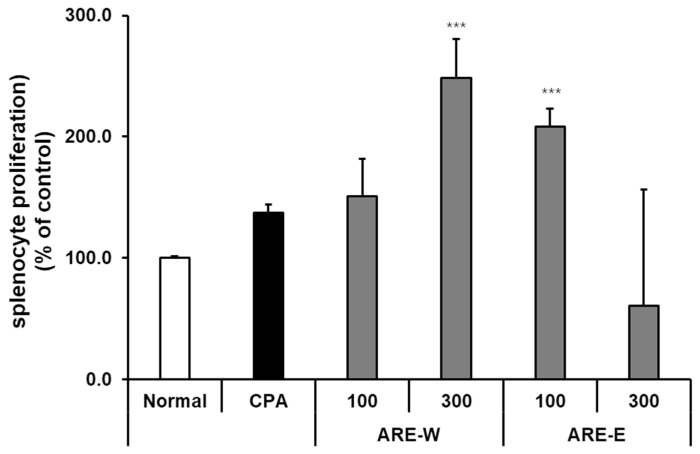
Effect of ARE-W and ARE-E on splenocyte proliferation in CPA-induced immunosuppressed mice. The CPA and ARE-W and ARE-E administration groups received intraperitoneal injections of cyclophosphamide (150 mg/kg, day −3; 110 mg/kg, day −1), followed by daily oral administration of ARE-W or ARE-E (100 or 300 mg/kg) for 14 days. The group treated with CPA alone served as the control group. Splenocytes isolated from different experimental groups were cultured for 72 h. Cell proliferation was determined using the EZ-Cytox assay and expressed as a percentage of the control (normal group). Data are presented as mean ± SD (*n* = 3). Statistical significance was determined by one-way ANOVA followed by Tukey’s post-hoc test. *** *p* < 0.001 versus the CPA group.

Lymphocyte proliferation capacity is an important indicator measuring the ability of immune cells to divide and proliferate in response to stimuli. In this study, we evaluated the effect of *A. rugosa* extract on splenic lymphocyte proliferation in a CPA-induced immunosuppressed mouse model using the EZ-Cytox assay. The results showed that there was no significant difference in splenic lymphocyte proliferation between the CPA group and the normal group. However, the ARE-W groups showed dose-dependent promotion of lymphocyte proliferation. Particularly, the 300 mg/kg group showed an approximately 2.4-fold increase compared to the normal group (*p* < 0.001) and a 1.9-fold increase compared to the CPA group (*p* < 0.001). The ARE-E groups showed the highest proliferation-promoting effect at a 100 mg/kg concentration (approximately 2.1-fold compared to the normal group, *p* < 0.001), while at a 300 mg/kg concentration, the effect was diminished, showing an inverse dose-dependent pattern (Figure 5). These results indicate that *A. rugosa* extract can effectively enhance splenic lymphocyte proliferation in immunosuppressed states. This effect may contribute to the recovery of T and B cell immune responses, and as the administration of ARE-W showed significant effects in both innate immunity (NK cell activity and IFN-γ production) and adaptive immunity (lymphocyte proliferation), it can be predicted to have a broad immunomodulatory effect across the overall immune system.

## 3. Discussion

This study comprehensively evaluated the immunoenhancing effects of *A. rugosa* extract in a CPA-induced immunosuppressed mouse model. The results confirmed that *A. rugosa* extract can effectively restore immune function impaired by CPA, suggesting its potential value as a natural immunomodulator in immunosuppressed states.

CPA is an anticancer agent and immunosuppressant that inhibits cell proliferation through DNA alkylation. This leads to selective damage to rapidly dividing immune cells, resulting in overall immune function impairment. In this study, CPA administration induced a clear immunosuppressive state, including body weight reduction, spleen weight decrease, and NK cell activity impairment, consistent with previous studies [34,35].

*A. rugosa* extract demonstrated various improvements in immune parameters in this immunosuppressed state. Particularly notable results were the increases in NK cell activity and IFN-γ production. NK cells are key components of innate immunity that directly recognize and eliminate virus-infected and tumor cells, and recovery of their function implies enhancement of antiviral and antitumor immunity. ARE-W (300 mg/kg) restored CPA-impaired NK cell activity to above-normal levels and increased IFN-γ production approximately 3-fold. IFN-γ strengthens immune responses through mechanisms such as macrophage activation and increased MHC molecule expression [36,37], suggesting that *A. rugosa* extract may positively influence both innate and adaptive immunity.

Recovery of spleen weight and lymphocyte proliferation capacity are also important findings. The spleen is a major immune organ, and recovery of its size and function implies normalization of the overall immune system. *A. rugosa* extract significantly increased CPA-reduced spleen weight and remarkably enhanced lymphocyte proliferation capacity. This suggests that *A. rugosa* extract can contribute to the structural and functional recovery of immune organs by promoting immune cell survival and proliferation. The difference in efficacy according to the extraction method is an interesting finding. In most immune parameters, ARE-W showed superior effects compared to ARE-E, particularly in NK cell activity and lymphocyte proliferation. This suggests that water-soluble components with immunoenhancing activity might be more efficiently extracted during the hot water extraction process. *A. rugosa* contains various flavonoids, phenolic acids, and essential oil components, among which certain water-soluble components are likely key contributors to the immunoenhancing effect, though further research is needed to identify which specific components are primarily responsible.

Based on existing literature, the observed gastroprotective effects of *A. rugosa* can be attributed to several key bioactive compounds: Tilianin and Acacetin, previous studies have demonstrated that these flavonoids exhibit significant anti-inflammatory properties [38,39]. Tilianin has been shown to provide neuroprotection through anti-inflammatory and anti-apoptotic pathways, while acacetin demonstrates neuroprotective properties by inhibiting oxidative damage and neuroinflammation [23,39]. Rosmarinic acid, a phenolic compound, has been identified as one of the main components of *A. rugosa* extracts and has been demonstrated to enhance therapeutic properties [21].

From a dose-dependence perspective, the 300 mg/kg concentration was more effective than 100 mg/kg in most parameters, but in some parameters (IFN-γ production by ARE-E, lymphocyte proliferation), the lower dose showed superior effects. This indicates that different immune parameters have distinct optimal dosing requirements. In contrast, ARE-W exhibited consistent dose-dependent improvements across all immune parameters, suggesting that extraction method influences both bioactive compound composition and optimal dosing profiles. These findings highlight that ARE-E requires parameter-specific dose optimization, whereas ARE-W demonstrates straightforward dose-response relationships. This emphasizes the importance of extraction method selection in determining both biological activity and optimal dosing strategy.

These findings have significant clinical implications for managing immunosuppressive conditions arising from cancer treatment, organ transplantation, and autoimmune disease therapy. The comprehensive immune enhancement demonstrated by ARE-W, improving both innate and adaptive immunity, makes it a promising candidate for clinical applications requiring broad-spectrum immune support, such as vaccine adjuvant development or treatment of immunocompromised patients. This suggests its potential application as a natural immunomodulator in conditions such as cancer therapy, autoimmune disease treatment, or age-related immune decline. For clinical translation, ARE-W shows potential as an immune-supporting therapy for chemotherapy patients and elderly individuals with weakened immunity.

However, several limitations must be acknowledged. This study was conducted in a single murine model, and validation in other species and immunosuppression models is essential. Additionally, long-term safety profiles, identification of active compounds, and dose optimization require further investigation. Future studies on active component identification, molecular mechanism investigation, and clinical efficacy and safety assessment are anticipated to facilitate the development of *A. rugosa*-based immunoenhancing materials. Human dose studies, safety testing, and component standardization are needed before clinical use.

## 4. Materials and Methods

### 4.1. Preparation of Agastache rugosa Extract

In this study, *A. rugosa* was collected from Seongsan-eup, Seogwipo-si, Jeju Island (coordinates: 33°27′47.5″ N, 126°54′48.9″ E, altitude 17 m). Taxonomic identification was performed by Professor Jun-Ho Song of Chungbuk National University, and the plant specimen was confirmed as *A. rugosa*. For ARE-W preparation, 100 g of dried *A. rugosa* aerial parts was pulverized and mixed with 1 L of purified water, then subjected to hot water extraction for 2 h. The extract was filtered and concentrated using a rotary evaporator (Rotavapor R-100, Buchi, Switzerland). The concentrated extract was then freeze-dried (IlshinBio Co., Ltd., Dongducheon-si, Gyeonggi-do, Republic of Korea) to obtain the ARE-W sample. For ARE-E, the same amount of *A. rugosa* powder (100 g) was extracted with 1 L of 20% ethanol solution (*v*/*v*) under reflux conditions at 30 °C for 2 h. After extraction, the filtered solution was concentrated under reduced pressure and freeze-dried to ensure complete removal of ethanol residues and freeze-dried using the same method to produce the final ARE-E.

### 4.2. Experimental Animals

Seven-week-old male BALB/c mice were acclimated for one week in an environment with a controlled light/dark cycle (12 h, 9 a.m. to 9 p.m., light/dark) before use in experiments. Feed (a commercial standard rodent diet, Supplementary Figure S1) and water were provided ad libitum. The number of animals per group (n = 4) was determined based on institutional ethical guidelines. All animal experiments were approved by the Institutional Animal Care and Use Committee of Gachon University (approval number: GU1-2021-IA0057-00/6 January 2022).

### 4.3. Reagents

For natural killer (NK) cell activity measurement, Serum Free Medium (SFM) and penicillin/streptomycin (Gibco, Bleiswijk, The Netherlands), and fetal bovine serum (Atlas, Fort Collins, CO, USA) were used. Roswell Park Memorial Institute (RPMI) 1640 medium and Phosphate-Buffered Saline (PBS) solution buffer (Corning Inc., Corning, NY, USA) were used for YAC-1 cell culture, and an NK isolation kit (130-115-818, Miltenyi Biotec, Bergisch Gladbach, Germany) was used for NK cell isolation. A Tumor Necrosis Factor (TNF)-α kit (555268, BD, Franklin Lakes, NJ, USA) and Interferon (IFN)-γ kit (DY485-05, R&D, Minneapolis, MN, USA) were used for measuring splenic lymphocyte cytokines. Concanavalin A (Con A) was purchased from Sigma Aldrich (St. Louis, MO, USA), and EZ-Cytox (EZ-3000, DoGenBio, Seoul, Republic of Korea) and EZ-LDH kits (EZ-LDH500, DoGenBio, Seoul, Republic of Korea) were used for cell viability and toxicity measurements. For animal experiments, a 0.5 *w*/*v* methylcellulose 400 (CMC) solution (Wako, Tokyo, Japan) and cyclophosphamide (CPA) (Tokyo Chemical Industry, Tokyo, Japan) as an immunosuppressant were used.

### 4.4. Evaluation of Immune-Restoring Effects of Jeju ARE in Cyclophosphamide-Treated Mice

**Figure 6:**
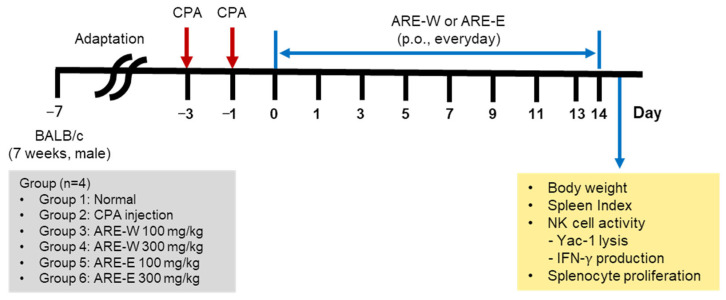
Experimental design for cyclophosphamide-induced immunosuppression and ARE treatment in mice.

For the immunosuppression experiment, mice were randomly divided into six groups (n = 4): (1) normal group, (2) CPA control group, (3) CPA + ARE-W 100 mg/kg group, (4) CPA + ARE-W 300 mg/kg group, (5) CPA + ARE-E 100 mg/kg group, and (6) CPA + ARE-E 300 mg/kg group. Except for the normal group, all mice received i.p. injections of CPA at 150 mg/kg (day −3) and 110 mg/kg (day −1) to induce immunosuppression. From day 0 to day 13, the normal and CPA groups received daily oral administration of CMC solution, while the extract-treated groups received daily oral administration of ARE-W or ARE-E at doses of 100 mg/kg and 300 mg/kg, respectively. Body weight was measured every 2 days, and mice were sacrificed on day 14 to collect spleens (Figure 6).

### 4.5. Body Weight and Spleen Index Measurement

Body weight was measured at 2-day intervals throughout the experimental period. On the day after the final oral administration on day 14, mice were sacrificed and spleens were excised and weighed. The spleen index was calculated as follows: spleen index (mg) = spleen weight (mg)/body weight (g).

### 4.6. Analysis of Tumor Cell Cytotoxicity by NK Cells

After sacrificing mice from each group, spleens were excised and processed with PBS using a stainless-steel mesh to obtain splenic cells. NK cells were isolated from splenocytes using an NK cell isolation kit (Miltenyi Biotec, Bergisch Gladbach, North Rhine-Westphalia, Germany) according to the manufacturer’s instructions. Isolated NK cells were adjusted to a concentration of 1 × 10^5^ cells/well and used as effector cells. NK cell cytotoxicity was measured using the lactate dehydrogenase (LDH) assay method. NK cells (effector cells) and YAC-1 cells (target cells) were co-cultured at ratios of 5:1 and 10:1 (E:T ratio) in round-bottomed 96-well microplates and incubated at 37 °C, 5% CO_2_ for 6 h. After incubation, the supernatant was collected and the LDH activity was measured using an EZ-LDH kit. NK cell cytotoxicity against tumor cells was calculated according to the following formula:

Lysis (%) = [(Experimental release − Spontaneous release)/(Maximum release − Spontaneous release)] × 100

### 4.7. Analysis of IFN-γ Production by NK Cells

NK cells isolated as described in Section 4.6 (1 × 10^5^ cells/well) were co-cultured with YAC-1 cells, and the supernatant was collected after 20 h. IFN-γ concentration in the supernatant was measured using a Mouse IFN-gamma ELISA kit (R&D Systems, Minneapolis, MN, USA) according to the manufacturer’s instructions.

### 4.8. Analysis of Splenic Lymphocyte Proliferation

Splenic cells were isolated from the spleens of mice after completion of oral administration, red blood cells were removed, and cells were seeded at 4 × 10^5^ cells/well in 96-well plates. After 72 h of incubation at 37 °C, 5% CO_2_, EZ-Cytox was added, and the plates were incubated for an additional 60 min before measuring absorbance at 450 nm.

### 4.9. Statistical Analysis

Experimental results were statistically analyzed using GraphPad Prisma 5.02, and the mean and standard deviation (SD) were calculated for all measurement parameters. Statistical analysis of data was performed using one-way ANOVA, and significant differences between groups were determined by Tukey’s post-hoc test. Results are expressed as * *p* < 0.05, ** *p* < 0.001, and *** *p* < 0.0001 to indicate statistical significance between groups.

## 5. Conclusions

The results of this study demonstrate that *A. rugosa* extract, particularly ARE-W, can effectively restore immune function in CPA-induced immunosuppressed states. ARE-W demonstrated comprehensive immune enhancement by improving both innate and adaptive immunity, including NK cell activity, IFN-γ production, spleen weight recovery, and lymphocyte proliferation. These findings suggest the potential application of *A. rugosa* extract as a natural immunomodulator for managing immunosuppressive conditions. However, further studies on active component identification, molecular mechanisms, and clinical safety assessment are needed before clinical application.

## Data Availability

The original contributions presented in the study are included in the article, further inquiries can be directed to the corresponding author.

## Supplementary Materials

The following supporting information can be downloaded at: https://www.mdpi.com/article/10.3390/ph18081129/s1, Figure S1: Full nutritional composition of Rodent Diet.

## Abbreviations

The following abbreviations are used in this manuscript:

CPA	Cyclophosphamide
NK	Natural killer
IFN-γ	Interferon-gamma
ARE-W	Hot water extract
ARE-E	Ethanol extract
Con A	Concanavalin A
i.p.	Intraperitoneal
LDH	Lactate dehydrogenase
SD	Standard deviation
