# Supplementary material for: Immunoenhancing Effects of Jeju Agastache rugosa Extracts on NK Cell Activity and Lymphocyte Proliferation in Cyclophosphamide-Induced Immunosuppression
Source: Pharmaceuticals (Basel). 2025 Jul 28;18(8):1129. doi: 10.3390/ph18081129 (PMC12389359; doi:10.3390/ph18081129)
Supplement: Supplementary file 1 [file pharmaceuticals-18-01129-s001.zip › pharmaceuticals-3666312-supplementary.pdf]

Supplementary Figure S1.

Full nutritional composition of Rodent Diet

PicoLab® Rodent Diet 20 — 5053

DESCRIPTION

PicoLab® Rodent Diet 20 is formulated with 20% protein diet and 4.5% fat. It is designed for rat, hamster and mouse breeding colonies. This diet is a complete life cycle diet formulated using managed formulation, delivering Constant Nutrition®. This is paired with the selection of highest quality ingredients to assure minimal inherent biological variation in long-term studies. Irradiated in 3-ply packaging to provide bioburden reduction for animals in a barrier facility. LabDiet® 5053 is offered as an irradiated pellet or meal; extruded and/or non-irradiated/autoclavable options also available.

Features and Benefits

- Managed Formulation delivers Constant Nutrition®
- High quality animal protein added to create a superior balance of amino acids for optimum performance
- Recommended for rat breeding colonies and mice not requiring a high energy diet
- Irradiation gives reliable microbial control and eliminates the need for autoclaving

Product Forms Available	*Catalog #*
• Oval pellet, (3/8"x5/8"x1"), Irradiated, 30 lb	3005740-220
• Meal (ground pellets), Irradiated, 30 lb	3005740-020
Other Irradiated Versions Available	***Catalog #***
• 5R53: PicoLab® Rodent Diet 20 Extruded, 20 lb	3002890-712
• 5R53: PicoLab® Rodent Diet 20 Extruded, Meal, 30 lb	3005839-020
• 5061: Pico-Vac® Lab Rodent Diet, Pelleted, 5 lb vacuum sealed, 6 per box, 30 lb	0006954
• 5K75: Certified PicoLab® Rodent 20, Pelleted, 30 lb	3005965-220
• 5LU7: PicoLab® Macro-Pack™ Rodent 20 75G, Pelleted, 15kg	0066400
Non-Irradiated Versions Available	***Catalog #***
• 5L0B: Laboratory Rodent Diet 20, Pelleted, 15 kg	0067097
• 5R53: Rodent Diet 20 Extruded, 15 kg	3002890-748
• 50A3: Autoclavable Rodent 20 Pelleted, 30 lb	3007163-446
• 5RA3: Autoclavable Rodent 20 Extruded, 25 lb	3006933-703

GUARANTEED ANALYSIS

Crude protein not less than	20.00%
Crude fat not less than	4.50%
Crude fiber not more than	6.00%
Ash not more than	7.00%
Moisture not more than	12.00%

INGREDIENTS

Ground Corn, Dehulled Soybean Meal, Wheat Middlings, Ground Wheat, Fish Meal, Dried Plain Beet Pulp, Cane Molasses, Wheat Germ, Brewers Dried Yeast, Ground Oats, Dehydrated Alfalfa Meal, Soybean Oil, Dried Whey, Calcium Carbonate, Salt, DL-Methionine, Menadione Dimethylpyrimidinol Bisulfite (Vitamin K), Choline Chloride, Pyridoxine Hydrochloride, Cholecalciferol (Vitamin D3), Vitamin A Acetate, DL-Alpha Tocopheryl Acetate (Vitamin E), Folic Acid, Thiamine Mononitrate, Manganous Oxide, Vitamin B12 Supplement, Zinc Oxide, Ferrous Carbonate, Nicotinic Acid, Riboflavin Supplement, Calcium Pantothenate, Copper Sulfate, Zinc Sulfate, Calcium Iodate, Cobalt Carbonate, Biotin, Sodium Selenite.

FEEDING DIRECTIONS

Feed ad libitum to rodents. Plenty of fresh, clean water should be available to the animals at all times.

Rats- All rats will eat varying amounts of feed depending on their genetic origin. Larger strains will eat up to 30 grams per day. Smaller strains will eat up to 15 grams per day. Feeders in rat cages should be designed to hold two to three days supply of feed at one time.

Mice-Adult mice will eat up to 5 grams of pelleted ration daily. Some of the larger strains may eat as much as 8 grams per day per animal. Feed should be available on a free choice basis in wire feeders above the floor of the cage.

Hamsters-Adults will eat up to 14 grams per day.

For information regarding shelf life please visit www.labdiet.com.

CHEMICAL COMPOSITION[1]

Nutrients[2]	
**Protein, %**	**21.0**
Arginine, %	1.29
Cystine, %	0.36
Glycine, %	0.98
Histidine, %	0.53
Isoleucine, %	0.87
Leucine, %	1.58
Lysine, %	1.18
Methionine, %	0.62
Phenylalanine, %	0.92
Tyrosine, %	0.61
Threonine, %	0.79
Tryptophan, %	0.24
Valine, %	0.97
Serine, %	1.00
Aspartic Acid, %	2.23
Glutamic Acid, %	4.26
Alanine, %	1.20
Proline, %	1.32
Taurine, %	0.03
**Fat (ether extract), %**	**5.0**
**Fat (acid hydrolysis), %**	**6.3**
Cholesterol, ppm	135
Linoleic Acid, %	2.32
Linolenic Acid, %	0.28
Arachidonic Acid, %	0.02
Omega-3 Fatty Acids, %	0.42
Total Saturated Fatty Acids, %	0.77
Total Monounsaturated Fatty Acids, %	1.00
**Fiber (Crude), %**	**4.4**
Neutral Detergent Fiber[3], %	15.5
Acid Detergent Fiber[4], %	5.6
**Nitrogen-Free Extract (by difference), %**	**53.5**
Starch, %	28.2
Sucrose, %	2.71
**Total Digestible Nutrients,%**	**75.1**
**Gross Energy, kcal/gm**	**4.11**
**Physiological Fuel Value[5], kcal/gm**	**3.43**
**Metabolizable Energy, kcal/gm**	**3.02**

Minerals	
**Ash, %**	**6.0**
Calcium, %	0.81
Phosphorus, %	0.61
Phosphorus (non-phytate), %	0.33
Potassium, %	1.07
Magnesium, %	0.21
Sulfur, %	0.31
Sodium, %	0.30
Chloride, %	0.53
Fluorine, ppm	9.2
Iron, ppm	184
Zinc, ppm	79
Manganese, ppm	82
Copper, ppm	13
Cobalt, ppm	0.72
Iodine, ppm	0.97
Chromium (added), ppm	0.01
Selenium, ppm	0.37

Vitamins	
Carotene, ppm	1.5
Vitamin K, ppm	3.3
Thiamin, ppm	16
Riboflavin, ppm	8.1
Niacin, ppm	84
Pantothenic Acid, ppm	17
Choline, ppm	1575
Folic Acid, ppm	3.0
Pyridoxine, ppm	9.6
Biotin, ppm	0.30
B_{12}, mcg/kg	51
Vitamin A, IU/gm	15
Vitamin D_3 (added), IU/gm	2.3
Vitamin E, IU/kg	99
Ascorbic Acid, mg/gm	0.00

Calories provided by:	
Protein, %	24.495
Fat (ether extract), %	13.122
Carbohydrates, %	62.382

1. Formulation based on calculated values from the latest ingredient analysis information. Since nutrient composition of natural ingredients varies and some nutrient loss will occur due to manufacturing processes, analysis will differ accordingly.
2. Nutrients expressed as percent of ration except where otherwise indicated. Moisture content is assumed to be 10.0% for the purpose of calculations.
3. NDF = approximately cellulose, hemi-cellulose and lignin.
4. ADF = approximately cellulose and lignin.
5. Physiological Fuel Value (kcal/gm) = Sum of decimal fractions of protein, fat and carbo- hydrate (use Nitrogen Free Extract) x 4,9,4 kcal/gm respectively.

NOTE: When assayed, actual levels may vary from calculated values.

LabDiet®
www.labdiet.com

09/05/23 RHI-W 29 (bag)
